# Impact of Illness and Medical Expenditure on Household Consumptions: A Survey in Western China

**DOI:** 10.1371/journal.pone.0052928

**Published:** 2012-12-28

**Authors:** Kuangnan Fang, Yefei Jiang, BenChang Shia, Shuangge Ma

**Affiliations:** 1 Department of Statistics, School of Economics, Xiamen University, Xiamen, China; 2 Department of Statistics and Information Science, FuJen Catholic University, New Taipei City, Republic of China; 3 School of Public Health, Yale University, New Haven, Connecticut, United States of America; Old Dominion University, United States of America

## Abstract

**Background:**

The main goal of this study is to examine the associations between illness conditions and out-of-pocket medical expenditure with other types of household consumptions. In November and December of 2011, a survey was conducted in three cities in western China, namely Lan Zhou, Gui Lin and Xi An, and their surrounding rural areas.

**Results:**

Information on demographics, income and consumption was collected on 2,899 households. Data analysis suggested that the presence of household members with chronic diseases was not associated with characteristics of households or household heads. The presence of inpatient treatments was significantly associated with the age of household head (p-value 0.03). The level of per capita medical expense was significantly associated with household size, presence of members younger than 18, older than 65, basic health insurance coverage, per capita income, and household head occupation. Adjusting for confounding effects, the presence of chronic diseases was negatively associated with the amount of basic consumption (p-value 0.02) and the percentage of basic consumption (p-value 0.01), but positively associated with the percentage of insurance expense (p-value 0.02). Medical expenditure was positively associated with all other types of consumptions, including basic, education, saving and investment, entertainment, insurance, durable goods, and alcohol/tobacco. It was negatively associated with the percentage of basic consumption, saving and investment, and insurance.

**Conclusions:**

Early studies conducted in other Asian countries and rural China found negative associations between illness conditions and medical expenditure with other types of consumptions. This study was conducted in three major cities and surrounding areas in western China, which had not been well investigated in published literature. The observed consumption patterns were different from those in early studies, and the negative associations were not observed. This study may complement the existing rural studies and provide useful information on western Chinese cities.

## Introduction

Illness conditions can be expensive. Multiple studies have suggested that medical expense may have a profound impact on other types of consumptions [1], [2], [3], [4]. The simple rationale is that with a limited budget, when facing medical expense, individuals and households may have to reduce consumptions of food, education, farming expense, other production means, recreation and others [5], [6]. Such reduction may have both short and long term impact.

China has the world’s largest population and the second largest economy by nominal GDP. In the recent years, there have been a large number of empirical studies on the ill health conditions, health insurance, and medical expenditure in China [7], [8], [9]. Our literature review suggested that the existing literature had been mainly focused on the distribution of illness conditions [10], distribution of medical expenditure and its associated factors [11], utility of health services [12], health insurance coverage and its impact on expenditure [13], and a few other topics. Although it is well acknowledged that medical expenditure may have an impact on consumption patterns, few studies have investigated such an impact in China [5].

A recent study was conducted by Nguyen and others in rural Vietnam [14] and investigated the impact of medical expense on consumption patterns. In that study, the mean per capita income was calculated as $630. It was found that households with inpatient treatments and higher levels of outpatient treatments had significantly decreased consumptions of basic capabilities such as food, education and production means. Setboonsarng and Lavado [6] made similar observations in a study conducted in rural Thailand, where the mean total household expenditure per year was about $1,723. The most relevant study was conducted by Wang and others [5], which reported a community-based survey conducted in poor rural areas of China in 2002. It was found that “medial expenditure reduced household investment in human capital, physical capital for farm production, and other consumptions that are critical to human well-being” [5].

The main goal of this study is to investigate the associations between illness conditions and medical expenditure with other types of consumptions, which differs from that in most of the aforementioned studies. The goal and study strategy are similar to those in [5], [14]. The differences from [5] include the selection of study subjects. In [5], data were collected from six small towns in poor, rural areas. In contrast, in this study data were collected from three major cities in western China and their surrounding areas. With a significant percentage of rural population, particularly new working-class, migrating to cities, large cities and their surrounding areas are of considerable interest. In addition, more household information was collected, providing a more comprehensive account for possible confounding effects. More importantly, with per capita GDP growing at about 10% annually during the past decade, China has been experiencing significant economic growth. Such growth has a direct impact on health care, medical expenditure, and consumption patterns. Thus, sensible differences are expected between [5] and the present study.

## Methods

### Data Collection

**Figure 1:**
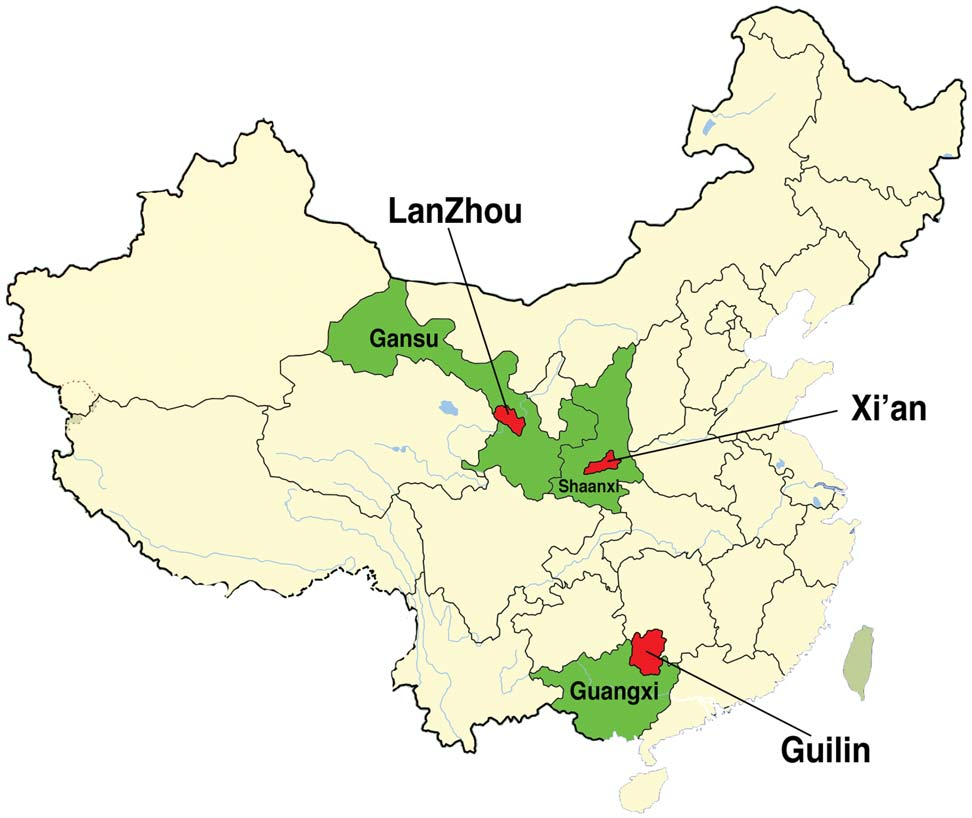
Map of the three surveyed cities.

The study was approved by a research ethics review committee at Xiamen University, China. Three cities in western China, namely Lan Zhou, Gui Lin and Xi An, and their surrounding rural areas were surveyed (Figure 1). Lan Zhou is the capital and largest city of GanSu Province. The population was about 3.6 million according to the 2010 census, and the per capita GDP was 25,566 RMB in 2008 (NBS GDP Data [15]; 1 US dollar = 6.35RMB in November 2011). Gui Lin is in the Guangxi Zhuang Autonomous Region. The population was about 4.7 million (2010 census), and the per capita GDP was 19,435RMB in 2009. Xi An is the capital of ShanXi Province. The population was about 8.5million in 2010, and the per capita GDP was 26,259RMB. As a comparison, the per capita GDP for the whole mainland China was 23,708RMB in 2008 and 25,608RMB in 2009. The three cities were chosen as representatives of large, sub-provincial cities in western China.

The survey was conducted by staff at the Data Mining Research Center (DMRC), Xiamen University, China, in November and December, 2011. A Computer-Assisted Telephone Survey System (CATSS) was adopted. The collection of phone numbers was purchased from China Telecom Corp. Ltd. and China Unicom Corp. Ltd. Samples were collected using an RDD (random digit dialing) approach. More specifically, Mitofsky-Waksberg [16] type samples of active blocks of 100 consecutive phone numbers were drawn from all possible such blocks within each city. The probability of a block’s initial selection was proportional to the block’s 100 numbers that served residences. The study database was updated after each phone call to ensure that no household was sampled multiple times. As it was difficult to associate a cell phone number with a physical location, this study focused on landlines only.

At the beginning of each survey, the staff would collect information to determine inclusion. A household would be excluded if (1) the interviewee refused to participate, (2) the household was not officially in the three surveyed cities, defined by “Hukou” (a household registration issued by the central government), (3) the interviewee was less than 18 years old, or (4) the interviewee could not provide reliable information on the household (self-evaluation). Verbal consent was obtained for each survey, recorded using voice recording software, and stored at DMRC. The survey included “snapshot” questions (such as demographic information, insurance status) as well as “accumulation” questions (such as income and expense over a period of twelve months prior to survey). On average, one survey took eight minutes. The survey response rate was about 39%.

### Statistical Analysis

Data was deidentified prior to analysis. Various graphical methods were employed to examine data, and no obvious outlier was found. The distributions of illness conditions (both chronic disease and inpatient treatment) and medical expenditure were first examined. Their associations with demographic variables were analyzed. Differences between different illness/medical expense groups were examined using t-tests for continuous variables and chi-squared tests for categorical variables. Similar techniques were used in the univariate analysis of illness conditions and consumption. Multivariate analyses were conducted to investigate the associations between illness conditions and medical expenditure with consumption patterns, adjusting for confounding effects. Here to get a more comprehensive description, we analyzed each consumption category separately, following the same strategy as in [5], [6]. Two sets of analyses were conducted. In the first set, the actual amount of consumption was analyzed using linear regression. In the second set, the percentage of each category of consumption (as of the total consumption) was analyzed using logistic-type regression. Model diagnostics was conducted, and no serious deviation from the model conditions was observed. Analysis was conducted using S-Plus Version 8.2 (TIBCO Software Inc.).

## Results and Discussion

The study collected data on 2,899 households (Table 1). The sample size was decided mainly based on resource availability. As household remained the basic functional unit for financial decisions in China, data was collected at the household level. This strategy was consistent with [5], [11]. In analysis, to account for the difference in household size, per capita income and expense were computed and analyzed.

**Table 1:** Sample characteristics of the whole cohort and subgroups with different illness conditions and medical expense levels.

		Wholecohort	Presence of chronic disease		Presence of inpatient treatment		Medical expense	
Variables			Yes	No	Yes	No	High	Low
Total sample		2899	1247	1652	1693	1206	1445	1454
LanZhou		954	450	504	506	388	448	506
GuiLin		979	401	578	582	397	497	482
XiAn		966	396	570	545	421	500	466
p-value			0.007		0.32		0.085	
**Household characteristics**								
Household size		4.84 (1.50)	4.84 (1.50)	4.84 (1.50)	4.84 (1.50)	4.84 (1.50)	5.05 (1.57)	4.66 (1.40)
p-value			0.79		0.82		<0.001	
Younger than 18*	0	19.52	20.21	19.01	19.37	19.73	17.72	21.32
	1	59.95	59.5	60.29	60.72	58.87	58.62	61.28
	2	17.73	17.32	18.04	17.31	18.33	19.93	15.54
	3	2.28	2.41	2.18	2.19	2.4	3.18	1.38
	4	0.38	0.32	0.42	0.3	0.5	0.35	0.41
	5	0.1	0.16	0.06	0.06	0.17	0.14	0.07
	6	0.03	0.08	0	0.06	0	0.07	0
p-value			0.79		0.78		<0.001	
Older than 65*	0	64.68	63.11	65.86	64.44	65.01	59.86	69.46
	1	18.59	19.73	17.74	19.02	17.99	17.92	19.26
	2	14.18	14.27	14.1	13.76	14.76	17.92	10.45
	3	1.35	1.68	1.09	1.59	1	2.08	0.62
	4	0.48	0.48	0.48	0.53	0.41	0.76	0.21
	5	0.52	0.48	0.54	0.47	0.58	1.04	0
	6	0.17	0.24	0.12	0.18	0.17	0.35	0
	7	0.03	0	0.06	0	0.08	0.07	0
p-value			0.58		0.71		<0.001	
Basic health insurance*		91.53 (17.48)	91.34 (17.88)	91.68 (17.17)	91.69 (17.23)	91.31 (17.83)	90.68 (18.17)	92.38 (16.73)
p-value			0.6		0.57		0.009	
Commercial health insurance*		83.41 (21.93)	83.53 (21.89)	83.32 (21.96)	82.82 (21.88)	82.84 (21.99)	82.67 (22.45)	84.15 (21.37)
p-value			0.8		0.24		0.07	
Hukou*	Urban	65.16	65.12	65.19	65.09	65.26	66.3	64.03
	Rural	34.84	34.88	34.81	34.91	34.74	33.7	35.97
p-value			0.99		0.96		0.21	
Income (RMB)		7341 (5347)	7156 (5392)	7480 (5310)	7304 (5321)	7391 (5384)	10851 (5001)	3852 (2781)
p-value			0.11		0.67		<0.001	
**Household head characteristics**								
Age*	<20	1.93	2.17	1.76	1.71	2.24	1.8	2.06
	21–30	8.07	7.78	8.29	8.68	7.21	7.96	8.18
	31–40	2.93	3.05	2.85	2.95	2.9	2.63	3.23
	41–50	37.22	36.17	38.01	35.91	39.05	36.4	38.03
	51–60	38.36	38.41	38.32	37.8	39.14	40.28	36.45
	>60	11.49	12.43	10.77	12.94	9.45	10.93	12.04
p-value			0.65		0.03		0.39	
Gender*	Male	75.82	75.3	76.21	76.2	75.29	76.82	74.83
p-value			0.60		0.60		0.23	
Education*	< = middle school	48.57	47.47	49.39	48.08	49.25	48.37	48.76
	High school	40.63	40.82	40.5	40.87	40.3	40.07	41.2
	Bachelor	9.14	9.94	8.54	8.98	9.37	9.83	8.46
	>Bachelor	1.66	1.76	1.57	2.07	1.08	1.73	1.58
p-value			0.52		0.21		0.61	
Marital status*	Single	24.35	24.22	24.46	24.1	24.71	24.22	24.48
	Married	62.99	63.03	62.95	63.85	61.77	63.25	62.72
	Divorced	6.28	6.5	6.11	5.67	7.13	6.64	5.91
	Widowed	6.38	6.26	6.48	6.38	6.38	5.88	6.88
p-value			0.97		0.39		0.62	
Occupation*	Government	17.45	16.68	18.04	17.54	17.33	28.44	6.53
	State-owned Company	21.18	21.17	21.19	20.73	21.81	37.37	5.09
	Private Company	5.35	4.81	5.75	5.2	5.56	10.17	0.55
	Self-employed	18.73	21.17	16.89	18.84	18.57	2.28	35.08
	Farmer	30.94	29.43	32.08	30.42	30.27	21.18	40.65
	Unemployed	1.97	1.76	2.12	2.13	1.74	0.07	3.85
	Retired	3.31	3.69	3.03	3.07	3.65	0.42	6.19
	Other	1.07	1.28	0.91	1.06	1.08	0.07	2.06
p-value			0.076		0.95		<0.001	

Values are means (standard deviations) and percentages (for variables marked by *).

According to [17] in 2011, for the GanSu province, the per capita income was 14989RMB for urban and 3909RMB for rural areas; For the GuangXi Zhuang Autonomous Region, the per capita income was 18854RMB for urban and 5231RMB for rural; For the ShanXi province, the average per capita income was 18245RMB for urban and 5028RMB for rural. In our survey, for the whole cohort (with about 65% urban and 35% rural households), the per capita income was 7341RMB, with standard deviation 5347RMB. The income data suggested that the surveyed samples might provide reasonable information for the three provinces/autonomous region. It is further noted that for the whole mainland China, the per capita income was 21810RMB for urban and 6977RMB for rural. The surveyed western areas had considerably lower income than national average, as expected. Summary statistics for other variables were scarce in the literature.

### Characteristics of Illness Conditions and Medical Expenditure

In Table 1, summary statistics of household and household head characteristics were presented for the whole cohort and subgroups characterized by different illness conditions and levels of medical expense. Illness condition was described using two variables: presence of (members with) chronic diseases, which were long-term, with multiple episodes and low cost per episode, and presence of (members with) inpatient treatments, which were low-frequency, high-cost health shocks. Medical expense was defined as the per capita, out-of-pocket medical expense accumulated over a period of twelve months prior to survey. In this study, we focused on the out-of-pocket medical expense, which, compared with gross medical expense, might better represent financial burden to households and individuals. Medical expense was a continuous variable. In Table 1, it was dichotomized at the median to create “high” and “low” cost groups.

Cross-city difference was observed, with LanZhou reporting more households with chronic diseases (p-value 0.007). Otherwise, the presence of chronic disease was not significantly associated with household or household head characteristics. The presence of inpatient treatment was significantly associated with the age of household head (p-value 0.03). For example, when the household heads were younger than 20, 43% of the households reported inpatient treatments, while this percentage was 58% when the household heads were older than 60. However no significant linear trend was observed. Analysis of per capita medical expense suggested that larger households (p-value<0.001), a larger number of members younger than 18 (p-value <0.001), a larger number of members older than 65 (p-value <0.001), lower basic health insurance coverage (p-value 0.009), higher per capita income (p-value <0.001), and household heads working for government or state-owned or private companies, were associated with higher medical expense.

Many of the above results had intuitive interpretations. The positive association between household size and per capita medical expense was relatively new and interesting. Wang and others [5] and Nguyen and others [14] also collected information on household size, however did not examine its association with medical expense. The association between medical expense and household head occupation could be partly explained by the association between occupation and income (in China, government, state-owned company, and private company employees tended to have higher income) and differences in basic health insurance systems for people with different occupations [11].

### Associations between Illness Conditions and Consumption

In the survey, consumption was measured in nine categories (Table 2). Each category was defined as the per capita consumption accumulated over a period of twelve months prior to survey. In our preliminary study, it was found that some households might purchase food, daily goods, clothes, and other items together, and had trouble separating those expenses (details omitted here). Thus, unlike in [5], the category of “basic consumption” was created to include food, clothes, production means, utilities and daily goods. More details were provided in Table 2. Basic consumption was the biggest category, accounting for 31.45% of the total consumption. Other major consumptions included saving and investment (23.67%), medical expense (15.54%), and insurance (15.39%). All other categories combined accounted for 13.95% of the total consumption. The observed consumption patterns differed significantly from those in some of the existing studies. For example, in [5], the percentage of medical expense was considerably lower (7.9%), the percentage of education was considerably higher (12.0%), and the percentage of saving was much lower (3.8%). In [14], the Vietnam study, medical expense accounted for 5.9% of the total expense, insurance only accounted for 0.2%, there was no separate category for saving, but the “other” category (which presumably included saving) accounted for only 4.6%. In [6], the Thailand study, medical expense accounted for only 2% of the total expense, education accounted for 16%, and there was no separate category for saving or other. Multiple factors might contribute to the observed differences, including for example the significant difference in total consumption (2043.4RMB per household in [5], compared to 8284.9RMB per capita in this study), geographic differences, and rural-urban differences. It should be noted that households surveyed in this study had a much higher saving rate, which might reflect their higher financial status. Households with regular savings might be able to cope with medical expense without having to lower daily living standard [11].

**Table 2:** Per capita expense (mean and standard deviation) and percentage (as of the total expense) for the whole cohort and subgroups with different illness conditions and medical expense levels.

	Whole cohort	Presence of chronic disease		Presence of inpatient treatment		Medical expense	
		Yes	No	Yes	No	High	Low
**Amount of expense (RMB)**							
Basic (food, produce, etc)	2884.0	2741.1	2991.9	2854.1	2926.1	4426.3	1351.3
sd	2397.4	2332.0	2440.8	2377.6	2425.3	2382.7	1051.2
p-value		0.005		0.43		<0.001	
Education	462.6	451.2	471.3	461.6	464.0	732.3	194.6
sd	386.6	389.2	384.6	389.1	383.3	353.6	172.5
p-value		0.17		0.87		<0.001	
Saving/Investment	2710.3	2097.8	2225.0	2169.3	2171.6	3346.5	1001.4
sd	1713.0	1688.2	1729.9	1727.8	1692.6	1602.1	747.2
p-value		0.05		0.97		<0.001	
Entertainment	465.8	449.2	478.3	463.7	468.7	736.0	197.3
sd	394.7	387.4	399.8	396.6	392.3	367.9	177.6
p-value		0.05		0.74		<0.001	
Insurance	1410.9	1376.0	1437.2	1391.1	1438.6	2183.2	643.4
sd	1176.6	1174.9	1177.6	1161.1	1198.0	1148.1	518.2
p-value		0.17		0.29		<0.001	
Durable goods	245.0	232.2	254.7	242.6	248.3	398.6	92.3
sd	242.5	234.9	247.8	239.0	247.4	244.7	105.1
p-value		0.01		0.53		<0.001	
Alcohol/Tobacco	101.5	97.6	104.4	101.2	101.9	175.4	28.0
sd	119.9	119.5	120.1	120.7	118.7	123.2	52.3
p-value		0.13		0.87		<0.001	
Other	4.8	7.5	2.7	4.0	6.0	6.4	3.2
sd	58.2	74.6	41.8	43.0	74.6	75.5	33.0
p-value		0.04		0.40		0.13	
**Percentage of expense**							
Basic (food, produce, etc)	31.45	31.03	31.75	31.33	31.62	30.92	33.32
Education	5.04	5.11	5.00	5.07	5.04	5.11	4.80
Saving/Investment	23.67	23.75	23.61	23.81	23.47	23.37	24.69
Entertainment	5.08	5.09	5.08	5.09	5.07	5.14	4.87
Insurance	15.39	15.58	15.25	15.27	15.55	15.25	15.87
Durable goods	2.67	2.63	2.70	2.66	2.68	2.78	2.28
Alcohol/Tobacco	1.11	1.10	1.11	1.11	1.10	1.23	0.69
Other	0.05	0.09	0.03	0.04	0.06	0.04	0.08

* Basic consumption includes food (rice, meet, vegetable, fruit, etc), clothes, production means (e.g. farming equipment, fertilizer, seed, etc), utilities (electricity, water, heating, cooking, renting, etc), and daily goods (toiletries, kitchen supplies); Education includes tuition, book, and other education-related expenses; Saving/investment includes banking, stock; Entertainment includes entertainment, travel, holidays and other social activities; Insurance: for property, farm product, health, etc; Durable goods include furniture and electronic devices; Alcohol/Tobacco: cigarette, tobacco, wine, liqueur; Medical expense: outpatient and inpatient services, drugs; Other: expenses not listed above.

The associations between illness conditions and consumption patterns were investigated using univariate analysis (Table 2) and multivariate analysis (Table 3). As multivariate analysis could be more informative, all main conclusions were based on Table 3. In multivariate analysis, possible confounding effects of household and household head characteristics were accounted for (details presented in Table 3). Two sets of analyses were conducted. In the first set, the actual amount of each category of expense was regressed on illness conditions and confounders, using linear regression models. In the second set, the percentage of each category of expense (as of the total expense) was analyzed. As the response variables were percentages between 0 and 1, logistic-type regression analysis was conducted [5], [18]. As the number of households with “other expense” was small and the actual amount was small, this expense category was not analyzed.

**Table 3:** Multivariate analysis of associations between presence of chronic disease and inpatient treatment with expense, measured by both the actual amount and percentage.

	Amount of expense				Percentage of expense			
	Presence of chronic disease		Presence of inpatient treatment		Presence of chronic disease		Presence of inpatient treatment	
	Est.	p-value	Est.	p-value	OR	p-value	OR	p-value
Basic (food, produce, etc)	−119.30	0.02	−47.80	0.34	0.96	0.01	0.98	0.18
Education	2.34	0.72	1.60	0.81	1.02	0.10	1.01	0.26
Saving/Investment	−30.51	0.31	14.34	0.64	1.00	0.70	1.02	0.17
Entertainment	−6.63	0.33	−1.32	0.85	1.01	0.36	1.01	0.66
Insurance	0.55	0.98	−37.19	0.13	1.04	0.02	0.98	0.19
Durable goods	−9.71	0.06	−3.33	0.51	0.99	0.58	1.00	0.81
Alcohol/Tobacco	−1.19	0.66	0.75	0.78	1.01	0.74	1.01	0.60

* Adjusted for household characteristics (household size, presence of younger than 18, presence of older than 65, basic insurance coverage, commercial insurance coverage, per capita income, city, Hukou) and household head characteristics (age, gender, education, occupation, marital status). Est: estimated regression coefficient in linear regression. OR: odds ratio in logistic regression.

Table 3 suggested that with other factors being equal, compared to households without inpatient treatment, households with inpatient treatments had a lower level of basic consumption (estimated difference = 119.3RMB, p-value 0.02). The association with consumption of durable goods was borderline significant (p-value 0.06). The associations with other consumptions were not significant. The presence of inpatient treatment was not significantly associated with any consumption. In the analysis of expense percentage, it was found that the presence of chronic disease was significantly negatively associated with the percentage of basic consumption (odds ratio 0.96, p-value 0.01) and positively associated with the percentage of insurance expense (odds ratio 1.04, p-value 0.02). Other associations were not significant. The presence of inpatient treatment was not associated with the percentages of consumptions.

### Associations between Medical Expense and Other Expenses

The associations between medical expense and other types of expenses were also directly studied. More specifically, other types of expenses were regressed on medical expense, adjusting for possible confounding effects (details presented in Table 4). As with illness conditions, two sets of analyses were conducted, one on the actual amount of consumption and the other on percentage.

**Table 4:** Multivariate analysis of associations between medical expense and other household consumptions, measured by the actual amount and percentage.

	Amount of expense		Percentage of expense	
	Est.	p-value	OR	p-value
Basic (food, produce, etc)	0.75	<0.001	0.67	<0.001
Education	0.13	<0.001	1.00	0.77
Saving/Investment	0.53	<0.001	0.90	<0.001
Entertainment	0.13	<0.001	0.98	0.16
Insurance	0.35	<0.001	0.86	<0.001
Durable goods	0.08	<0.001	0.98	0.49
Alcohol/Tobacco	0.04	<0.001	0.98	0.54

* Adjusted for household characteristics (household size, presence of younger than 18, presence of older than 65, basic insurance coverage, commercial insurance coverage, per capita income, city, urban) and household head characteristics (age, gender, education, occupation, marital status). Est: estimated regression coefficient in linear regression. OR: odds ratio in logistic regression.

Table 4 suggested that medical expense was positively associated with all other types of expenses (all p-values<0.001). The largest estimated regression coefficient, which loosely speaking corresponded to the highest level of association, was for basic consumption with estimated coefficient 0.75. The analysis of percentage expense suggested that medical expense was negatively associated with basic expense (odds ratio 0.67, p-value<0.001), saving and investment (odds ratio 0.90, p-value<0.001), and insurance expense (odds ratio 0.86, p-value <0.001).

## Discussion

In the analysis of illness conditions, negative associations were observed between the presence of chronic disease and basic consumption and saving and investment. Such negative associations were also observed in [5], [14]. Associations with other consumption categories were not observed. With all other variables fixed, the amount of reduction in basic consumption (because of chronic disease) was only about 4.1% of the total consumption, which was considerably smaller than that observed in [5]. Such observations were in fact reasonable. China has experienced significant economic growth during the past decade, making households less vulnerable to health shocks. The total household consumption in rural China in [5] was 2043.4RMB (average household size unspecified), and that in Vietnam in [14] was 12517.5VND (740USD, approximately 4706.7RMB) for a household with 3.8 members on average. Households surveyed in this study had considerably higher overall consumption levels. In addition in our survey, on average 23.67% of household expense was saving and investment. With a higher financial status and a higher saving rate, households might not need to significantly reduce consumption to cope with medical expense. Table 2 suggested that the biggest consumption categories were basic consumption, saving and investment and insurance. In Table 3, basic consumption and saving and investment had the largest (negative) estimated coefficients. Other consumption categories were already small, leaving very little room for reduction. As discussed in [11] and references therein, commercial health insurance was not well developed in China, and the basic health insurance system had limited flexibility, with premium usually prefixed. This might explain why a significant decrease or increase in insurance expense was not observed.

In the analysis of medical expense (actual amount), the observed positive associations between medical expense and other expenses differed significantly from what was observed in [5], and may seem counterintuitive. The survey collected cross-sectional, observational data. It should be noted that as in many published studies, such data can only identify association, not causation [19]. In multiple publications, a positive association between out-of-pocket medical expense and income/expense has been observed [20]. Households with a higher total budget could have higher levels of expenses in multiple categories, leading to the observed positive associations. In China, the positive associations between medical expense and household income and total household consumption were recently reported in [11]. Compared to [14], medical expense accounted for a much smaller percentage of the total expense. Thus, change in medical expense was not necessarily correlated with a significant amount of change in other expenses. The discrepancy between this study and [5], [14] could also be explained by the argument offered in [21], which stated that the observation that poor households spending more on healthcare were typically based on small sample studies conducted in rural areas. The second set of analysis on expense percentage might better describe the scenario with a fixed budget, as the percentages summed to one. Here negative associations were observed between the percentage of medical expense and the percentages of other expenses. The most notable association arose from basic consumption (odds ratio 0.67). Such an observation was consistent with [5], [6] and other studies.

### Limitations

In this study, cross-sectional, observational data were collected. With such data, only associations could be concluded, not causality. To fully track the changes in non-medical expenses following illness conditions and quantify the causal effects of medical expense, longitudinal studies with more strictly controlled conditions may have to be conducted. It is noted that several studies that investigated the relationship between illness and consumption, such as [5], [14], adopted a similar study strategy and hence shared the same limitation. In this study, data was collected via phone call survey. With limited resources, phone call survey can generate a larger number of samples. However, the nature of phone call survey inevitably led to certain drawbacks. For example, only a limited number of variables could be collected, and potentially important factors could be missed in data collection. The set of selected variables had been suggested by multiple studies, and might include the most important ones. Interviewees were asked to recall the total amount of income and expense for a period of twelve months. It has been suggested that such an approach may lead to a biased estimation (usually under estimation) [22]. Without having access to a second source of data, such bias cannot be completely ruled out and corrected. The illness condition of a household was measured by the presence of chronic disease and inpatient treatment. Such measures did not take into account the types of illness and number of episodes. On the other hand, such seemingly crude measures had been shown to be reasonably informative in [5], [14] and others. The expense data reflected the aggregation over one year, which made it impossible to investigate the timing-dependent properties of consumption. All samples were collected from three large cities and their surrounding areas in western China. The GDP figures [15] and income data suggested that the survey samples could be representative of the three provinces/autonomous region, but not the whole mainland China. With resources available to collect only a limited number of samples, it was impossible to provide informative description of the whole China.

### Conclusions

Published studies had been mainly conducted in other Asian countries (mostly rural areas) and rural China. It was suggested that illness conditions and medical expenditure could be correlated with significant reductions in households’ basic capabilities. Survey reported in this study was conducted in three large cities and their surrounding areas in western China. By studying a region previously less investigated, and by analyzing more recent data, this study may provide useful information beyond the existing studies. Although data analysis suggested certain associations between illness condition/medical expense and other consumptions, the observed associations were considerably different from those in published studies. Such results may suggest that the surveyed households were in a better position dealing with financial consequences of health shocks (compared to those in previous studies). However, studies that can better describe the causal effects (as opposed to simply association) are needed to verify such a statement.
